# Supplementary figures and images for: XPF-ERCC1 protects liver, kidney and blood homeostasis outside the canonical excision repair pathways
Source: PLoS Genet. 2020 Apr 9;16(4):e1008555. doi: 10.1371/journal.pgen.1008555 (PMC7144963; doi:10.1371/journal.pgen.1008555)

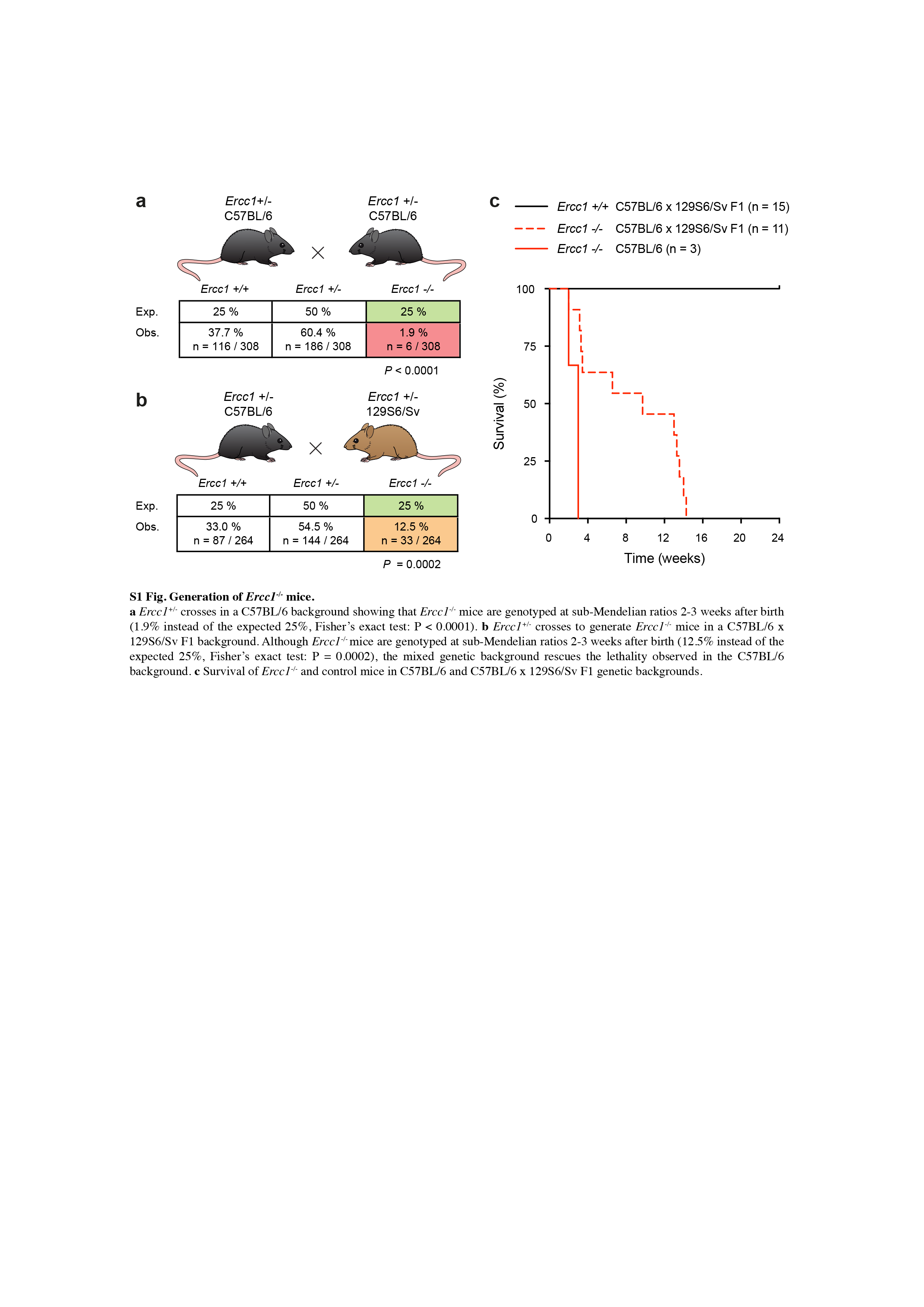

Supplement: S1 Fig — a) Ercc1+/- crosses in a C57BL/6 background showing that Ercc1-/- mice are genotyped at sub-Mendelian ratios 2–3 weeks after birth (1.9% instead of the expected 25%, Fisher’s exact test: P < 0.0001). b) Ercc1+/- crosses to generate Ercc1-/- mice in a C57BL/6 x 129S6/Sv F1 background. Although Ercc1-/- mice are genotyped at sub-Mendelian ratios 2–3 weeks after birth (12.5% instead of the expected 25%, Fisher’s exact test: P = 0.0002), the mixed genetic background rescues the lethality observed in the C57BL/6 background. c) Survival of Ercc1-/- and control mice in C57BL/6 and C57BL/6 x 129S6/Sv F1 genetic backgrounds. (TIF) [file pgen.1008555.s001.tif]

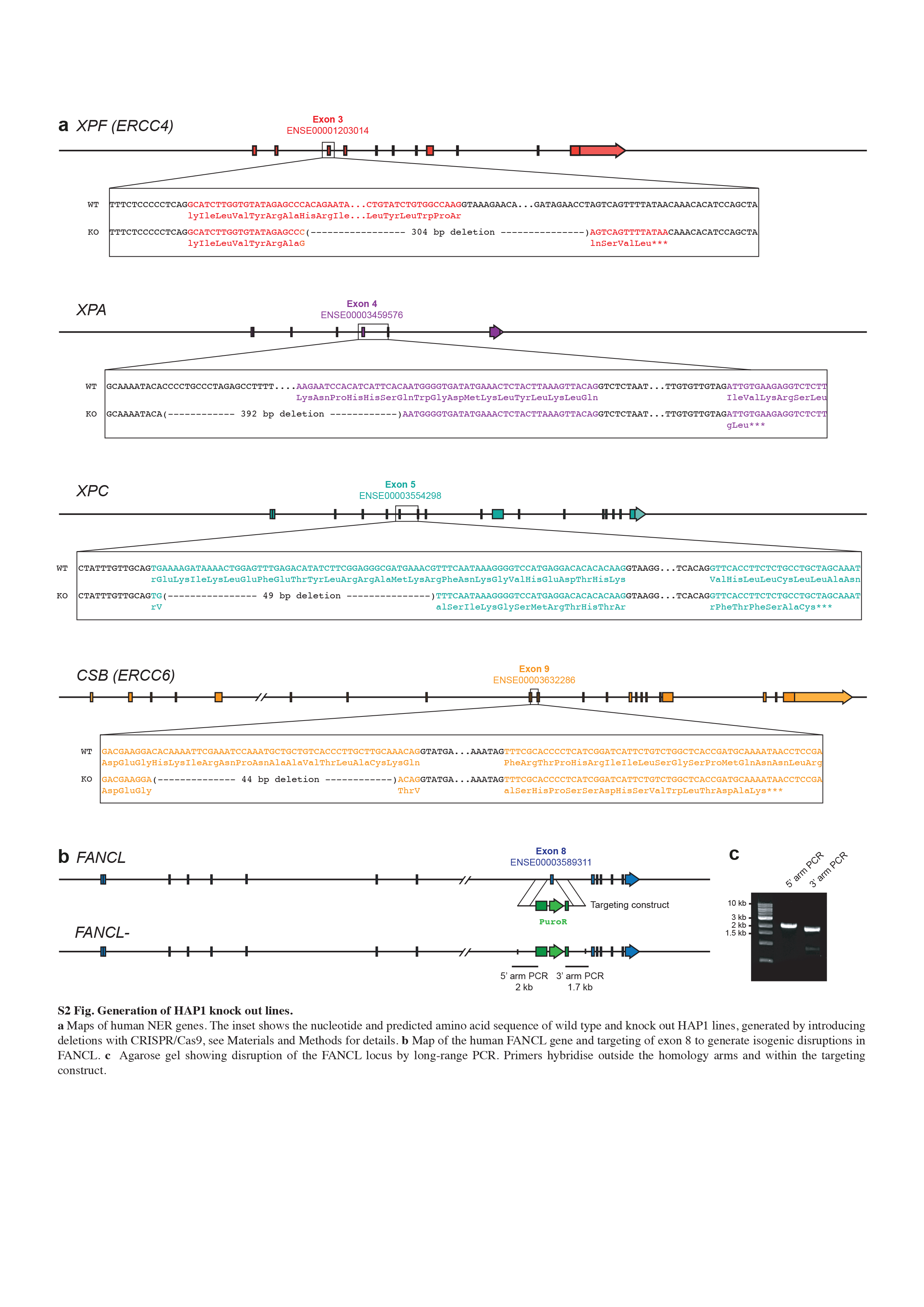

Supplement: S2 Fig — a) Maps of human NER genes. The inset shows the nucleotide and predicted amino acid sequence of wild type and knock out HAP1 lines, generated by introducing deletions with CRISPR/Cas9, see Methods for details. b) Map of the human FANCL gene and targeting of exon 8 to generate isogenic disruptions in FANCL. c) Agarose gel showing disruption of the FANCL locus by long-range PCR. Primers hybridise outside the homology arms and within the targeting construct. (TIF) [file pgen.1008555.s002.tif]

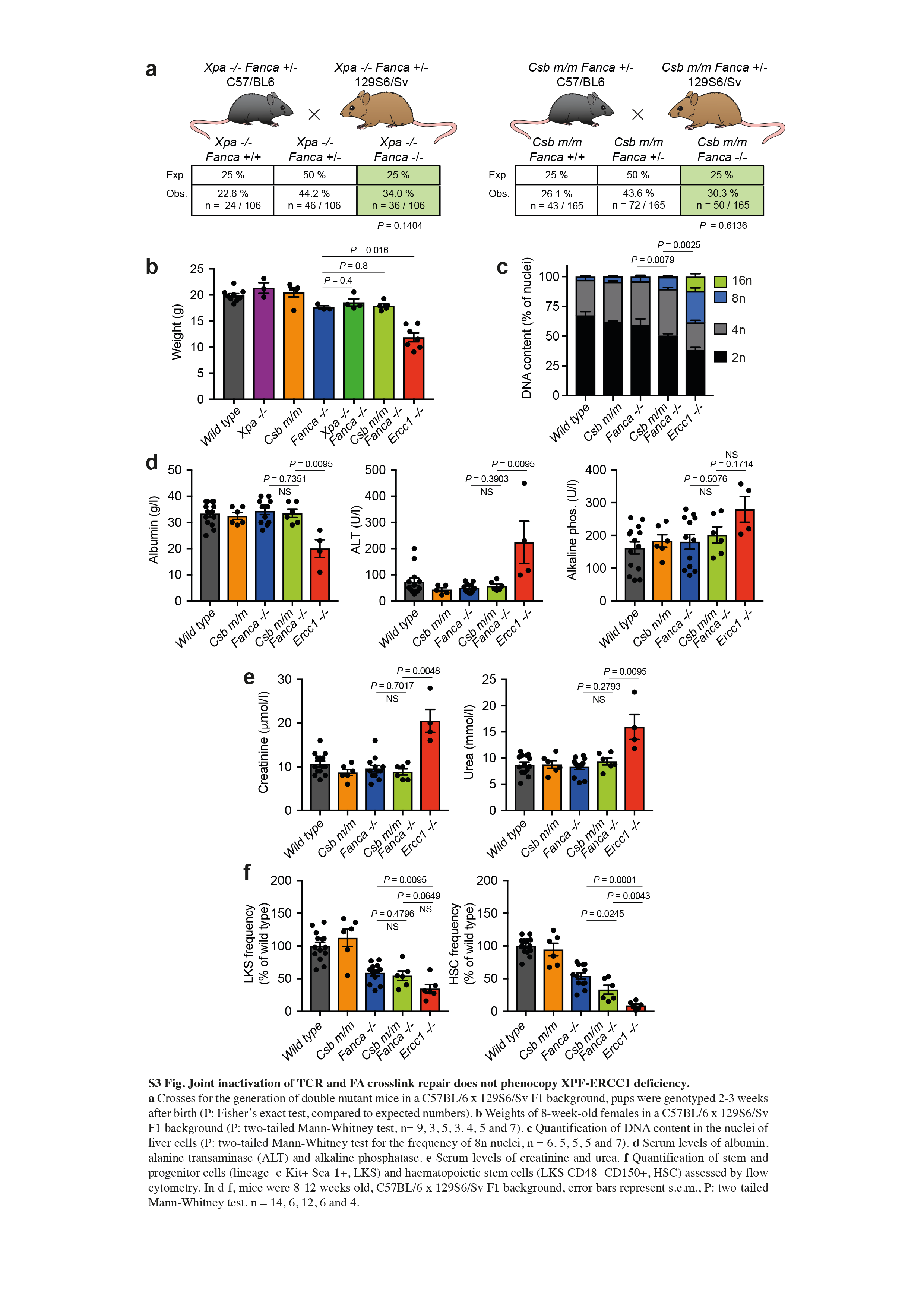

Supplement: S3 Fig — a) Crosses for the generation of double mutant mice in a C57BL/6 x 129S6/Sv F1 background, pups were genotyped 2–3 weeks after birth (P: Fisher’s exact test, compared to expected numbers). b) Weights of 8-week-old females in a C57BL/6 x 129S6/Sv F1 background (P: two-tailed Mann-Whitney test, n = 9, 3, 5, 3, 4, 5 and 7). c) Quantification of DNA content in the nuclei of liver cells (P: two-tailed Mann-Whitney test for the frequency of 8n nuclei, n = 6, 5, 5, 5 and 7). d) Serum levels of albumin, alanine transaminase (ALT) and alkaline phosphatase. e) Serum levels of creatinine and urea. f) Quantification of stem and progenitor cells (lineage- c-Kit+ Sca-1+, LKS) and haematopoietic stem cells (LKS CD48- CD150+, HSC) assessed by flow cytometry. In d-f, mice were 8–12 weeks old, C57BL/6 x 129S6/Sv F1 background, error bars represent s.e.m., P: two-tailed Mann-Whitney test. n = 14, 6, 12, 6 and 4. (TIF) [file pgen.1008555.s003.tif]

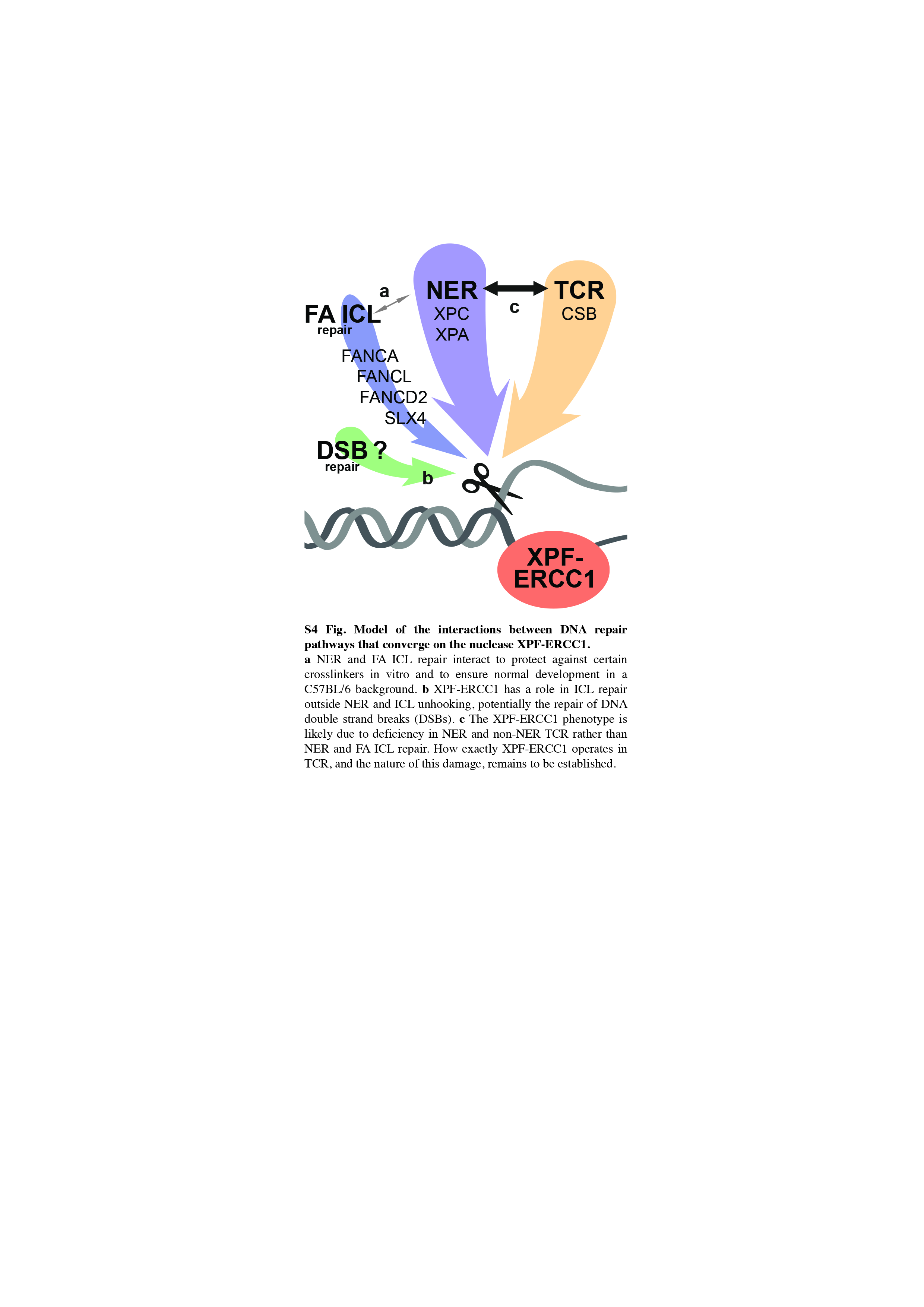

Supplement: S4 Fig — a) NER and FA ICL repair interact to protect against certain crosslinkers in vitro and to ensure normal development in a C57BL/6 background. b) XPF-ERCC1 has a role in ICL repair outside NER and ICL unhooking, potentially the repair of DNA double strand breaks (DSBs). c) The XPF-ERCC1 phenotype is likely due to deficiency in NER and non-NER TCR rather than NER and FA ICL repair. How exactly XPF-ERCC1 operates in TCR, and the nature of this damage, remains to be established. (TIF) [file pgen.1008555.s004.tif]
