# Supplementary material for: XPF-ERCC1 protects liver, kidney and blood homeostasis outside the canonical excision repair pathways
Source: PLoS Genet. 2020 Apr 9;16(4):e1008555. doi: 10.1371/journal.pgen.1008555 (PMC7144963; doi:10.1371/journal.pgen.1008555)
Supplement: S1 Table — (DOCX) [file pgen.1008555.s005.docx]

**Supplementary Table 1. gRNAs**

| **Gene** | **Left CRISPR** | **Right CRISPR** |
| --- | --- | --- |
| *XPC* | 5’ GTTTGAGACATATCTTCGGAGGG 3’ | 5’ CAACAAATAGTGAAAAATCTGGG 3’ |
| *XPA* | 5’ GAATCCACATCATTCACAATGGG 3’ | 5’ TTCACAATAAATTTAAGAGGTGG 3’ |
| *CSB (ERCC6)* | 5’ TTCAAATGCTTCCCCAGTACAGG 3’ | 5’ GAGAACTGCTCCATAAACACAGG 3’ |
| *XPF (ERCC4)* | 5’ GACAAGACTCGATTATTCTGTGG 3’ | 5’ TTTCGCCAGAAAAACAAACGTGG 3’ |
| *FANCL* | 5’ ACAGCACGCAGAATTGCATTAGG 3’ | 5’ TTTTTCTGGCTCAAGTACCCAGG 3’ |
