# Supplementary material for: XPF-ERCC1 protects liver, kidney and blood homeostasis outside the canonical excision repair pathways
Source: PLoS Genet. 2020 Apr 9;16(4):e1008555. doi: 10.1371/journal.pgen.1008555 (PMC7144963; doi:10.1371/journal.pgen.1008555)
Supplement: S2 Table — (DOCX) [file pgen.1008555.s006.docx]

**Supplementary Table 2. Screening by PCR**

| **Gene** | **Forward Primer** | **Reverse Primer** |
| --- | --- | --- |
| *XPC* | 5’ TTTGAGGCATGTAGGTGAGGTGT 3’ | 5’ CCACGCTGATCTCTTTCACTGTT 3’ |
| *XPA* | 5’ CTCCTCACCATCGCTTACTTCAG 3’ | 5’ GGAGATGGAGGTGAAAGAGGGTA 3’ |
| *CSB (ERCC6)* | 5’ AGTGTCCCCTTTTCTAAGTTTCTCC 3’ | 5’ CTACATTCGATTGATGCAGGATGAC 3’ |
| *XPF (ERCC4)* | 5’ TCCATCCAGCCTTTATTCAGATACC 3’ | 5’ TCAAAGCAGAAGTGGTAATGTTAGC 3’ |
| *FANCL* 5' arm | 5’ TTTTGGCAGGGGAGTTAACCTT 3’ | 5’ GCGATCTCTGGGTTCTACGTTAGTG 3’ |
| *FANCL* 3' arm | 5’ CACACCTCCCCCTGAACCTGAAAC 3’ | 5’ TAAGCCTTTCACTAAGGCTGGCA 3’ |
