# Supplementary material for: XPF-ERCC1 protects liver, kidney and blood homeostasis outside the canonical excision repair pathways
Source: PLoS Genet. 2020 Apr 9;16(4):e1008555. doi: 10.1371/journal.pgen.1008555 (PMC7144963; doi:10.1371/journal.pgen.1008555)
Supplement: S3 Table — (DOCX) [file pgen.1008555.s007.docx]

**Supplementary Table 3. Sanger sequencing of PCR products**

| **Gene** | **Forward Primer** | **Reverse Primer** |
| --- | --- | --- |
| *XPC* | 5’ TTTGCTGGTGAGAAGGAGC 3’ | 5’ GCAGCAAAGCCAGAAATAAAGC 3’ |
| *XPA* | 5’ CCTTTTTGCTGTGTGTGC 3’ | 5’ CACACTCTGTAAGCAAAAGCC 3’ |
| *CSB (ERCC6)* | 5’ ACCAATTTATGAGCCTGGCC 3’ | 5’ GCAGAGGAGCGTTTTAGGGT 3’ |
| *XPF (ERCC4)* | 5’ CTCTGTTCTGTGCGTGGCTA 3’ | 5’ CACATAATGTTCTTTACCTTGGCCA 3’ |
