# Supplementary material for: XPF-ERCC1 protects liver, kidney and blood homeostasis outside the canonical excision repair pathways
Source: PLoS Genet. 2020 Apr 9;16(4):e1008555. doi: 10.1371/journal.pgen.1008555 (PMC7144963; doi:10.1371/journal.pgen.1008555)
Supplement: Attachment — Submitted filename: Mulderrig - Response to Reviewers.pdf [file pgen.1008555.s008.pdf]

Reviewer's Responses to Questions

Comments to the Authors:
Please note here if the review is uploaded as an attachment.

Reviewer #1: Mulderrig and colleagues present a descriptive genetic study of the interaction between two key pathways thought to defend against DNA interstrand crosslinks (ICLs) in mammals, namely nucleotide excision repair (NER) and factors deficient in Fanconi anemia (FA). While little in the way of novel mechanism is revealed, much can be surmised from the detailed phenotypic analysis, and it is my view that this is an important and timely contribution. The reliance on the transcription-coupled aspects of NER (TC-NER) have not been previously addressed or demonstrated in a systematic fashion. The MS is well-written, the data support the conclusions, the experiments are relatively thorough and the work is generally of good quality.

We are pleased that the reviewer shares our enthusiasm for this work, and we thank him/her for the comments.

My only comments are as follows:

1. It has been suggested on a number of occasions that some rodent cell types are deficient, or at least show reduced, GG-NER, possibly due to low DDB2 levels, originally referred to as the 'rodent repairadox'. It remains unclear for many cell types whether this is really the case in vivo, but the possibility that certain rodent cells types could be less efficient in performing GG-NER than perhaps human cells are should be acknowledged and its ramifications briefly discussed.

We thank the reviewer for raising the issue of the 'rodent repairdox'. This term is used to describe the observation that human and rodent cells display similar clonal survival characteristics when exposed to UV light. However, cultured cells from mice, rats and hamsters are less efficient at excising clyclobutane pyrimidine dimers (CPDs, the main UV adduct) compared to human cells (reviewed in Hanawalt 2001). This effect is thought to be the consequence of reduced levels of DDB2 (also p48, or XPE) in rodent cells (Hwang et al. 1999). DDB2 is a component of the GG-NER pathway but, unlike XPC that detects a wide range of helix-distorting lesions independently of the nature of damage , DDB2 is an accessory NER subunit that specializes on the recognition of UV damage (Yeh et al. 2012; Fischer et al. 2011). While this is an interesting observation, and could potentially explain differences between rodent and human phenotypes, we respectfully disagree with reviewer and believe this is not directly relevant to our current manuscript for two reasons.

First, the 'rodent repairdox' has been described only in the case of UV damage, and specifically for CPDs, not 6-4 photoproducts (6-4PP). Without knowing if the same applies to other NER/bulky lesions, we cannot be sure that rodent cells have reduced or deficient GG-NER. If the difference in CPD excision between rodent and human cells is mainly due to the levels of DDB2, which specializes in the recognition of UV damage, then we predict that the 'rodent repairdox' will not hold up for the excision of endogenous lesions, which are most relevant for our mouse work. Our work focuses on the homeostasis of liver, kidney and bone marrow, and the DNA damage in these internal organs cannot be driven by UV light.

Second, here we use well-established mouse models of NER deficiency (*Xpc-/-*, *Xpa-/-*, *Csb m/m*). From these models, we know that GG-NER matters in mice because *Xpc-/-* are sensitive to UV radiation or DMBA treatment. The surprising result from our study is that *Xpa-/-Fanca-/-*, with complete absence of NER and ICL repair, do not show obvious phenotypes. But importantly, the comparison we are drawing is between *Xpa-/-Fanca-/-* and *Ercc1-/-* mice which display a severe phenotype, in the same C57BL/6 x 129S6/Sv genetic background. The 'rodent repairdox' would be more relevant if we were trying to compare our mouse phenotypes (or lack thereof) with human DNA repair deficiency but that is not the case.

In summary, we are cautious of extrapolating data on DNA repair responses to UV damage in cultured cells to the repair of endogenous lesions in a living organism. The differing efficiencies in the ability of GG-NER to excise UV lesions between mouse and human cells cannot explain why we

observe differences when comparing mouse models. We believe this addition would confuse the reader and divert from the main message from our paper: joint disruption of NER and ICL repair does not equal XPF-ERCC1 deficiency in mice.

2. In the Discussion section, few alternative suggestions are proposed for the identity of the XPF-ERCC1 dependent ICL repair pathway(s) that act in addition to TC-NER and the FA pathways. It would be nice to see the authors 'stick their necks out' and speculate a little on this point: could it/they be FAN1, SNM1A or mismatch repair-related?

We thank the reviewer for pointing this out, we have now modified the discussion to expand and speculate on this point (lines 363-372). We think that two possibilities could explain the greater hypersensitivity of *XPF-/-* cells compared to *XPA-/-FANCL-/-* cells in response to crosslinking agents.

In the first place, XPF-ERCC1 orthologues play an important role in the repair of DSBs and meiosis in flies, plants and yeast. In *S. cerevisiae*, the Rad1-Rad10 nuclease resolves DNA intermediates by removing the 3' flap of non-homologous sequence during single-strand annealing (SSA) and microhomology-mediated end-joining (MMEJ), and similar roles have been proposed for XPF-ERCC1 in the resolution of DSBs in mammals. So, it is possible that XPF-ERCC1 has a role in HR in the resolution of the 'normal' DSB generated following unhooking of the crosslink. Alternatively, XPF-ERCC1 could be part of a back-up mechanism that resolves the 'toxic' DSBs that are generated when unhooking fails. Therefore, in this scenario, while disruption of *FANCL* would only disrupt unhooking, disruption of *XPF* would disrupt unhooking together with a subsequent (or backup) repair step.

The second possibility is that XPF-ERCC1 acts in an entirely separate, FA-independent ICL repair pathway. Recently, the novel NEIL3 pathway has been identified, but this pathway relies on the glycosylase NEIL3 and does not necessitate incisions of the phosphodiester backbone. A number of other nucleases have been implicated in crosslink repair, including MUS81-EME1, SLX1, FAN1 and SNM1A. Interestingly, SNM1A is epistatic with XPF-ERCC1 for crosslink repair but non-epistatic with FANCC (Ishiai et al. 2004; Wang et al. 2011). This would indeed suggest that SNM1A acts independently of the FA pathway to repair ICLs, but in a common pathway with XPF-ERCC1. This is now discussed in lines 363-372.

Reviewer #2: Loss of the XPF/ERCC1 nuclease complex results in a more profound phenotype than absence of other nucleotide excision repair (NER) genes. Ercc1-/- mice are born at substantially less than expected frequencies and die shortly after birth. In humans, complete inactivation of either XPF or ERCC1 is incompatible with viability. Although the basis of the apparently absolute requirement for XPF/ERCC1 remains uncertain, it is known that the complex is important for multiple DNA transactions: NER (both global and transcription coupled), but also interstrand crosslink (ICL) repair via the Fanconi Anemia (FA) pathway. Additionally, ERCC1/XPF has a function in telomere maintenance, contributes to repair of double strand breaks in both homology directed and end joining pathways, and is involved in the repair of abasic lesions. Presumably the profound phenotype of XPF/ERCC1 deficiency reflects diminished efficacy of the collective of these, or as yet unidentified, pathways.

In an effort to clarify the basis for the extreme phenotype the authors of this submission have examined the response to various DNA damaging agents, including acetaldehyde, formaldehyde, cisplatin and MMC in cells either lacking ERCC1, or XPA (representing loss of both GG and TC-NER), or FANCL (a key FA gene), or both XPA and FANCL. XPA and the FANCL double knockout cells were not as sensitive to cisplatin, MMC, or acetaldehyde as ERCC1-/- cells. These results emphasized the greater pathology of Ercc1-/- as compared to loss of both NER pathways, or the FA pathway, or the combined pathways. They argued that Ercc1 performed repair functions additional to those of NER and FA. These results and conclusion contrasted with the results with formaldehyde treated cells in which the FANCL-/-XPA-/- cells were just as sensitive as the ERCC1-/- cells.

The authors also studied the effect of combined NER and FA deficiency on mouse development. Xpa-/- Fanca-/- C57BL/6 pups were born as infrequently as Ercc1-/- animals. This observation was further dissected by examining pup frequencies in Fanca-/- Xpc-/- or Fanca-/- Csb-/- C57BL/6. Csb (mutant in Cockayne Syndrome and required for TCR) was chosen because it restricted the deficiency to TCR. The absence of Xpc in the Fanca-/- background did not affect birth frequencies, while Csb deficiency reduced them by half, significant, but not as profound as the Ercc1-/- or Xpa-/- Fanca-/- reduction. This result focused attention on the requirement for TC-NER and FA in normal development, but implied that additional ERCC1 pathways were also important.

The analyses of Ercc1 knockout was also performed in F1 hybrids of C57BL/6 crossed with 129S6/Sv, a strain that suppresses the developmental severity of Ercc1 loss. The role of the NER and FA pathways in postnatal life was examined in the F1 hybrids. Xpa-/- Fanca-/- pups were born at normal frequencies and examination of liver, kidney, and hematopoietic tissues indicated that the Ercc1-/- animals were more severely compromised than either the Xpa-/-Fanca-/- or Csb-/-Fanca-/- animals.

In their Discussion the authors cite work from van der Pluijm et al, who studied Csb and Csb/Xpa deficient C57BL/6 mice, whose birth frequencies were reduced but not as severely as Ercc1-/- mice.

We would like to thank the reviewer for the thorough summary and critical reading of our manuscript.

Critique:
This paper presents a series of thoughtfully designed and detailed analyses to address the question: do the NER and FA pathways account for the activities of XPF/ERCC1? The answer seems to be: it depends on the specifics of the assay. This rather mixed conclusion, in a narrative that twists and turns, should be clarified with some additional data and discussion. Otherwise it seems to be two parallel reports-one describing results with one damaging agent and mouse strain that conclude the affirmative, while the other presents the contrary view based on assays with different damaging agents and mouse background.

1. In the experiments with HAP1 knockout cells the authors found that the XPA/ FANCL double KO cells were as sensitive to formaldehyde as the XPF KO. Consequently, the elements of the XPF pathway that conferred protection to formaldehyde were duplicated by NER and FA. In contrast, the sensitivity of XPF KO cells to MMC, cisplatin, and acetaldehyde was not completely captured by XPA/FANCL

KO. This implied that while these compounds generated adducts repaired by NER and FA, they also produce toxic structure (s), not formed by formaldehyde, that is/are resistant to NER and FA, but resolved by XPF. XPF/ERCC1 contributes to various forms of double strand break (DSB) repair. Recent work in yeast (Cell Cycle 16, 45, 2017) suggest an involvement in homologous recombination pathways in the repair of acetaldehyde damage. Sensitivity to crosslinking agents is a mark of many HR deficiencies. The authors raise the possibility of ERCC1 participation in the repair of DSBs in the context of the compound sensitivity experiments. Do cells treated with these compounds show evidence for DSBs, perhaps replication related, that formaldehyde treated cells do not? Some simple immunofluorescence analyses or comet assays would address this.

The reviewer raises an interesting point: why is it that we see a different genetic interaction for formaldehyde compared to other crosslinking agents, or compared to the closely related aldehyde acetaldehyde? Our interpretation of this result is that formaldehyde causes a different spectrum of lesions, and NER and the FA pathway could be redundant pathways to repair some of these. While we agree with the reviewer that this is a very interesting point, we are unsure that simple immunofluorescence analyses or comet assays suggested by the reviewer would address the nature of this difference. This is because both formaldehyde and acetaldehyde have been shown to lead to replication related DSBs, including of RAD51 foci, sister chromatid exchanges, fork stalling and replication stress and chromosomal breakage (Tacconi et al. 2017; Tan et al. 2017). We anticipate that all treatments used in our study would lead to an induction of these markers.

We believe that these assays are too crude to allow us to draw definitive conclusions about the nature of the DNA damage caused by aldehydes, and how this is repaired. The alternative approach that we are exploring is to introduce site specific aldehyde-DNA lesions into the *Xenopus* egg extract system, which allows us to monitor its repair with single nucleotide resolution, as well as the pathways involved (Knipscheer et al. 2009).

However, we agree with the Reviewer that the discrepancy between formaldehyde and other crosslinkers is an interesting point. We originally alluded to this in the results section but have now emphasized this point in the Discussion (lines 374-377).

2. The adverse effects of FA deficiencies are interpreted as reflecting the activity of the pathway in ICL repair. Although this is reasonable, there is a school of thought that adds sensitivity to inflammatory mediators as an important aspect of FA. This is a feature that might have profound consequences during development and should be mentioned.

Indeed there is a link between the FA pathway and proinflammatory cytokines, and we have written a Review trying to reconcile the evidence for this with the defect in DNA repair (Garaycoechea and Patel 2014). It is worth mentioning that since this Review was published, more FA genes have been discovered directly linking FA to a defect in ICL repair: *FANCR (RAD51)*, *FANCS (BRCA1)*, *FANCT (UBE2T)*, *FANCU (XRCC2)* and *FANCV (REV7)*. We speculate that the inflammatory cytokine response may be an important source of reactive aldehydes and have included this in the revised version of the manuscript (at lines 233-235).

However, we must be careful not to over emphasize the potential role of inflammatory cytokines. The Reviewer suggests that inflammatory cytokines may contribute to the abnormal development of *Fanca-/-* embryos, although there is no direct evidence showing that loss of inflammatory cytokines/signaling suppresses developmental defects in FA mouse models. However, if we assume for a moment that inflammatory cytokines do contribute to developmental defects of *Fanca-/-* pups, it is not clear to us how lack of NER could further potentiate this phenotype through an inflammatory mechanism, leading to abnormal development of *Xpa-/-Fanca-/-* C57BL/6 pups. The commonality between NER and FA proteins is DNA repair, and to our knowledge XPA does not have other functions outside of NER. To us, the simplest explanation is that simultaneous inactivation of two DNA repair pathways is what corrupts development, and suggesting that *Xpa-/-Fanca-/-* C57BL/6 pups are underrepresented due to the action of inflammatory cytokines would be too speculative at this point.

3. In C57BL/6 mice Xpa/Fanca KO recapitulates the low birth yields seen with Ercc1 KO mice. Consequently, it appears that the NER and (at least some aspect of) the FA pathways substitute for Xpf/Ercc1 during early mouse development. The authors discuss this and conclude that the combination of the TCR and FA pathways "preserve development". However, the decline in live births in the Csb/Fanca pups is not as great as in the Xpa/Fanca background. Is there another, non NER, role for XPA?

To our knowledge, a role of outside of NER for XPA has not been reported. Our interpretation of these results is that *Xpa-/-* mice lack both GG-NER and TC-NER whereas *Csb m/m* mice lack TC-NER but have intact GG-NER. Although we did not detect a genetic interaction between GG-NER and the FA pathway (as seen by the normal ratios of *Xpc-/-Fanca-/-* pups), it is well established that there is redundancy between GG-NER and TC-NER, at least for the repair of UV damage. Therefore, we believe *Xpa-/- Fanca-/-* C57BL/6 pups are worse off because they lack GG-NER, TC-NER and ICL repair, but *Csb m/m Fanca-/-* C57BL/6 pups only lack TCR and ICL repair. To clarify this point, we have added additional text to the discussion (lines 381-384).

4. The results of with C57BL/6 were confounded by the suppression of effect of the Xpa/Fanca knock out in the C57BL/6 x 129S6/Sv cross. The experiments with the crossed animals are more difficult to interpret, given the amelioration of phenotypes by unknown modifiers supplied by 129S6/Sv. The operating assumption with the F1 hybrid is that the "modifying" component does not affect the deficiency under study. For example, the results indicate that Ercc1 loss is more consequential than Xpa/Fanca for various tissue markers, in the F1. Is it possible that the 129S6/Sv strain supplies a suppressor of FA deficiency, making the Xpa/Fanca KO F1 strain still functionally Xpa-/- but with diminished influence of FA inactivity? Are the cells from the F1 "FA-/-" as defective in Fanconi pathway functions (like ICL repair) as C57BL/6 "FA-/- "? Pace et al demonstrated in a well known study (Science 2010) that Ku70 "corrupts DNA repair in the absence of the FA pathway". Loss of Ku reduced sensitivity to ICLs and chromosome instability. Does the 129S6/Sv strain contribute something like this to the F1?

We agree with the Reviewer that the suppression of developmental problems by the 129S6/Sv genetic background is indeed very interesting. The genetic background of inbred strains has long been known to dramatically affect mouse phenotypes (Sittig et al. 2016). In this case, the identity of the modifier loci would shed light on cellular and organismal responses to DNA damage, we now allude to this in the Discussion (lines 391-397). However, we would like to clarify three points:

First, *Fanca -/-* mice in the C57BL/6 x 129S6/Sv F1 background still develop an HSC defect, which is indistinguishable to the HSC defect in *Fanca -/-* C57BL/6 mice (Figure 1). This shows that the 129S6/Sv background suppresses developmental defects but does not rescue the HSC compartment, showing that the *Fanca -/-* C57BL/6 x 129S6/Sv F1 HSCs are as defective as the *Fanca -/-* C57BL/6 HSCs. Indeed, we allude to this in the discussion (lines 390-392) and now emphasized the point in lines 400-401.

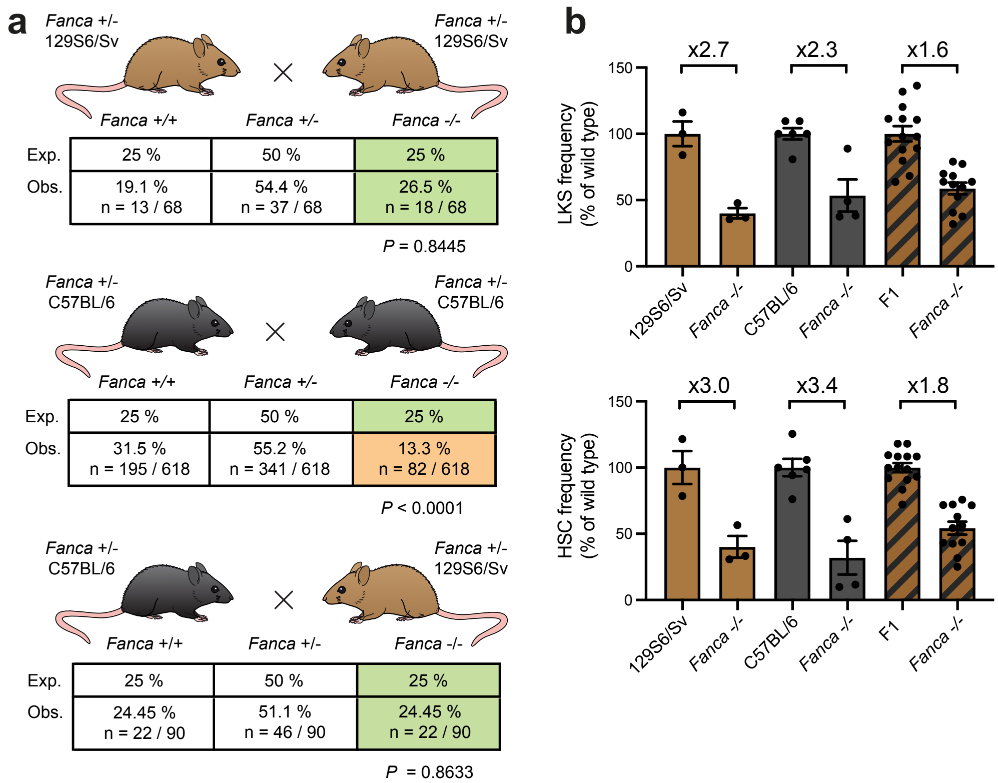

Figure 1. Genetic background affects development but not HSC defects of *Fanca-/-* mice. a) *Fanca*$^{+/-}$ crosses in the 129S6/Sv, C57BL/6 and 129S6/Sv x C57BL/6 F1 backgrounds. *Fanca*$^{-/-}$ mice are genotyped at sub-Mendelian ratios 2-3 weeks after birth in the C57BL/6 background (13.3% instead of the expected 25%, *P*: Fisher's exact test compared to expected numbers, but born at the expected Mendelian frequency in the 129S6/Sv and 129S6/Sv x C57BL/6 F1 backgrounds. **b)** Quantification of LKS (lineage- c-Kit+ Sca-1+) and haematopoietic stem cells (LKS CD48-CD150+, HSC) assessed by flow cytometry. The LKS and HSC defects are comparable in the three genetic backgrounds.

Second, the C57BL/6 background is susceptible to developmental defects like hydrocephalus and malocclusion (https://www.jax.org/news-and-insights/2003/july/hydrocephalus-in-laboratory-mice , https://www.jax.org/news-and-insights/2003/april/malocclusion-in-the-laboratory-mouse). More specifically, the potentiation of developmental defects in the C57BL/6 background is not unique to FA models, but actually commonly seen in models of DNA repair deficiency or other DNA repair pathways. This shows that any modifier loci present in the C57BL/6 background lead to developmental failure in response to DNA damage in general and are not specific to ICL repair. Some examples are listed below (and were also cited in the manuscript, lines 302-303):

Xpg	NER + TCR	(Barnhoorn et al. 2014)
Pole4	Replication	(Bellelli et al. 2018)
Atm	Damage signalling	(Genik et al. 2014)
Ku80	NHEJ	(Reliene et al. 2006)
Rev1	TLS	(Jansen et al. 2006)

Finally, the reviewer wonders if deficiency of NHEJ could explain the suppression of the FA developmental problems because work from our lab has previously shown that loss of Ku70 in chicken *FANCC-/-* DT40 cells rescues their survival in response to crosslinking agents (Pace et al. 2010). However, we know that these findings are not directly applicable to mice as we have shown that *Fanca-/-Ku70-/-* embryos are synthetically lethal and loss of Ku70 further potentiates HSCs defects in *Fanca fl/fl Ku70-/- Vav1-iCre+* mice (Garaycoechea et al. 2018; Bunting et al. 2012).

Therefore, we think that determining the identity of the modifier loci is an interesting avenue for future research, but beyond the scope of this work. Moreover, *Ercc1 -/-* mice in the C57BL/6 x 129S6/Sv F1 background develop liver, kidney and bone marrow dysfunction, and our experiments are

controlled as we compare their phenotype to *Xpa-/-*, *Fanca-/-* and *Xpa-/- Fanca-/-* mice in the exact same F1 genetic background.

1

5. In the end the authors reach the conclusion that the lack of TCR, of both NER and non NER lesions, accounts for much of the phenotype of XPF-ERCC1 deficiency. While that may make a central contribution, the results across the study are so influenced by experimental design so as to restrict conclusions to the specific conditions of the analysis.

We would like to clarify that we do not believe that lack of TCR alone that accounts for much of the phenotype of XPF-ERCC1 deficiency, because *Csb m/m* mice which lack TCR have a mild phenotype. Previous work with *Xpa-/-Csb m/m* and *Xpc-/-Csb m/m* have demonstrated that GG-NER and TCR have redundant functions for the repair of endogenous DNA damage, and these mice share phenotypic features with XPF-ERCC1 deficiency. When put together with our current work, we believe this points to a function of XPF-ERCC1 in non-NER TCR.

References

Barnhoorn S, Uittenboogaard LM, Jaarsma D, Vermeij WP, Tresini M, Weymaere M, Menoni H, Brandt RMC, de Waard MC, Botter SM, et al. 2014. Cell-autonomous progeroid changes in conditional mouse models for repair endonuclease XPG deficiency. *PLoS Genet* **10**: e1004686. http://www.ncbi.nlm.nih.gov/pubmed/25299392.

Bellelli R, Borel V, Logan C, Svendsen J, Cox DE, Nye E, Metcalfe K, O'Connell SM, Stamp G, Flynn HR, et al. 2018. Polε Instability Drives Replication Stress, Abnormal Development, and Tumorigenesis. *Mol Cell* **70**: 707-721.e7. http://www.ncbi.nlm.nih.gov/pubmed/29754823.

Bunting SF, Callén E, Kozak ML, Kim JM, Wong N, López-Contreras AJ, Ludwig T, Baer R, Faryabi RB, Malhowski A, et al. 2012. BRCA1 functions independently of homologous recombination in DNA interstrand crosslink repair. *Mol Cell* **46**: 125–35. http://www.ncbi.nlm.nih.gov/pubmed/22445484.

Fischer ES, Scrima A, Böhm K, Matsumoto S, Lingaraju GM, Faty M, Yasuda T, Cavadini S, Wakasugi M, Hanaoka F, et al. 2011. The molecular basis of CRL4 DDB2/CSA ubiquitin ligase architecture, targeting, and activation. *Cell* **147**: 1024–1039.

Garaycoechea JI, Crossan GP, Langevin F, Mulderrig L, Louzada S, Yang F, Guilbaud G, Park N, Roerink S, Nik-Zainal S, et al. 2018. Alcohol and endogenous aldehydes damage chromosomes and mutate stem cells. *Nature* **553**: 171–177. http://dx.doi.org/10.1038/nature25154.

Garaycoechea JI, Patel KJ. 2014. Why does the bone marrow fail in Fanconi anemia? *Blood* **123**: 26–34.

Genik PC, Bielefeldt-Ohmann H, Liu X, Story MD, Ding L, Bush JM, Fallgren CM, Weil MM. 2014. Strain background determines lymphoma incidence in Atm knockout mice. *Neoplasia* **16**: 129–36. http://www.ncbi.nlm.nih.gov/pubmed/24709420.

Hanawalt PC. 2001. Revisiting the rodent repairadox. *Environ Mol Mutagen* **38**: 89–96. http://www.ncbi.nlm.nih.gov/pubmed/11746740.

Hwang BJ, Ford JM, Hanawalt PC, Chu G. 1999. Expression of the p48 xeroderma pigmentosum gene is p53-dependent and is involved in global genomic repair. *Proc Natl Acad Sci U S A* **96**: 424–8. http://www.ncbi.nlm.nih.gov/pubmed/9892649.

Ishiai M, Kimura M, Namikoshi K, Yamazoe M, Yamamoto K, Arakawa H, Agematsu K, Matsushita N, Takeda S, Buerstedde J-M, et al. 2004. DNA cross-link repair protein SNM1A interacts with PIAS1 in nuclear focus formation. *Mol Cell Biol* **24**: 10733–41. http://www.ncbi.nlm.nih.gov/pubmed/15572677.

Jansen JG, Langerak P, Tsaalbi-Shtylik A, Van Den Berk P, Jacobs H, De Wind N. 2006. Strand-biased defect in C/G transversions in hypermutating immunoglobulin genes in Rev1-deficient mice. *J Exp Med* **203**: 319–323.

Knipscheer P, Räschle M, Smogorzewska A, Enoiu M, Ho TV, Schärer OD, Elledge SJ, Walter JC. 2009. The Fanconi anemia pathway promotes replication-dependent DNA interstrand cross-link repair. *Science* **326**: 1698–701. http://www.ncbi.nlm.nih.gov/pubmed/19965384.

Pace P, Mosedale G, Hodskinson MR, Rosado I V, Sivasubramaniam M, Patel KJ. 2010. Ku70 corrupts DNA repair in the absence of the Fanconi anemia pathway. *Science* **329**: 219–23. http://www.ncbi.nlm.nih.gov/pubmed/20538911.

Reliene R, Goad MEP, Schiestl RH. 2006. Developmental cell death in the liver and newborn lethality of Ku86 deficient mice suppressed by antioxidant N-acetyl-cysteine. *DNA Repair (Amst)* **5**: 1392–7. http://www.ncbi.nlm.nih.gov/pubmed/16916625.

Sittig LJ, Carbonetto P, Engel KA, Krauss KS, Barrios-Camacho CM, Palmer AA. 2016. Genetic Background Limits Generalizability of Genotype-Phenotype Relationships. *Neuron* **91**: 1253–1259. http://dx.doi.org/10.1016/j.neuron.2016.08.013.

Tacconi EM, Lai X, Folio C, Porru M, Zonderland G, Badie S, Michl J, Sechi I, Rogier M, Matía García V, et al. 2017. BRCA1 and BRCA2 tumor suppressors protect against endogenous acetaldehyde toxicity. *EMBO Mol Med* **9**: e201607446. http://embomolmed.embopress.org/lookup/doi/10.15252/emmm.201607446.

Tan SLW, Chadha S, Liu Y, Gabasova E, Perera D, Ahmed K, Constantinou S, Renaudin X, Lee MY, Aebersold R, et al. 2017. A Class of Environmental and Endogenous Toxins Induces BRCA2 Haploinsufficiency and Genome Instability. *Cell* **169**: 1105-1118.e15. http://dx.doi.org/10.1016/j.cell.2017.05.010.

Wang AT, Sengerová B, Cattell E, Inagawa T, Hartley JM, Kiakos K, Burgess-Brown NA, Swift LP, Enzlin JH, Schofield CJ, et al. 2011. Human SNM1A and XPF-ERCC1 collaborate to initiate DNA interstrand cross-link repair. *Genes Dev* **25**: 1859–70. http://www.ncbi.nlm.nih.gov/pubmed/21896658.

Yeh JI, Levine AS, Du S, Chinte U, Ghodke H, Wang H, Shi H, Hsieh CL, Conway JF, Van Houten B, et al. 2012. Damaged DNA induced UV-damaged DNA-binding protein (UV-DDB) dimerization and its roles in chromatinized DNA repair. *Proc Natl Acad Sci U S A* **109**: 2737–2746.
